# One-year patency control and risk analysis of eSVS®-mesh-supported coronary saphenous vein grafts

**DOI:** 10.1186/s13019-015-0293-y

**Published:** 2015-08-08

**Authors:** Devdas T. Inderbitzin, Jens Bremerich, Peter Matt, Martin T. R. Grapow, Friedrich S. Eckstein, Oliver Reuthebuch

**Affiliations:** 1 Department of Cardiac Surgery, University Hospital of Basel, Spitalstrasse 21, CH-4031 Basel, Switzerland; 2 Division of Radiology, University Hospital Basel, Petersgraben 4, CH-4031 Basel, Switzerland

**Keywords:** External saphenous vein meshing, Patency of coronary vein grafts

## Abstract

**Background:**

The eSVS® external venous nitinol mesh (Kips Bay Medical, Minneapolis, USA) was designed to improve long-term patency of coronary saphenous vein grafts (SVG) by preventing pressure-induced wall stress and reactive neo-intimal hyperplasia. We present one-year-patency rates of meshed SVGs assessed by coronary computed tomographic angiography (cCTA).

**Patients and Methods:**

Data from consecutive patients receiving an eSVS® meshed coronary bypass SVG from 06/2010 to 06/2011 were prospectively collected and analysed post-hoc. Patient characteristics, coronary artery disease, SVG quality, surgery (including number of anastomoses and transit time flow-measurement: TTFM), postoperative course and graft patency by cCTA were recorded. Potential risk factors for meshed graft occlusion were evaluated.

**Results:**

22 patients received an eSVS® mesh (18 isolated CABG, 4 combined with aortic valve replacement). Three patients died prior to the one-year follow-up and were excluded. All 19 surviving patients (mean age 70.4 ± 9.5 years, 3 female) completed a cCTA of all grafts at 12 ± 0.1 months after surgery including 21 meshed SVGs (33 distal anastomoses), 7 unmeshed SVGs (13 distal anastomoses) and 22 arterial grafts (30 distal anastomoses).

Mesh application was safe with patent grafts (by intraoperative TTFM) and perioperative course uneventful in all patients. The average graft/anastomosis number per patient was 2.6 ± 0.5/3.7 ± 0.8. Patency was unrestricted in all arterial and unmeshed SVGs (cCTA). Meshed SVG patency was 85 % (*n* = 28/33) for distal anastomoses and 76 % (*n* = 16/21) among meshed SVGs. Four SVGs with single distal anastomosis to the right coronary were completely occluded. One sequential graft to the left coronary was occluded between proximal and first distal anastomosis (see Fig. 1). Patency was independent of target site, coronary run-off, SVG quality and sequential distal grafting. All patients were asymptomatic.

**Conclusions:**

The overall one-year patency rate of eSVS® meshed SVGs/anastomoses was 76 %/85 %. Surgical implantation is safe independently of target site, run-off, vein quality and sequential distal anastomoses. However, graft patency of meshed veins (76 %) was inferior to non-meshed (100 %) or arterial grafts (100 %). Thus our mid-term data do not sustain the concept of improving vein graft patency by external reinforcing with the eSVS® mesh. Further long-term follow-up is warranted.

## Background

The use of autologous saphenous vein grafts (SVG) for coronary artery bypass grafting (CABG) has been adopted all over the world. However, intermediate- and long-term patency of SVGs is inferior to arterial grafts as to reactive intimal hyperplasia with luminal restriction and progressive atherosclerotic SVG disease with subsequent thrombotic graft occlusion [1, 2]. Despite the current tendency of favoring totally arterial revascularisation a lack of suitable graft material and the knowledge about favorable patency of SVGs in coronary stenosis < 90 % still make the use of SVGs essential [3, 4].

**Fig. 1:**
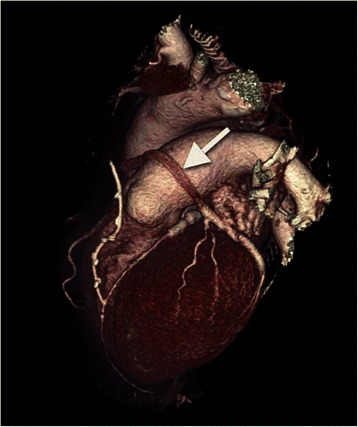
Coronary Computed Tomographic Angiography of a Partially Occluded Meshed SVG cCTA three-dimensional reconstruction showing a partially occluded eSVS® supported SVG between its proximal aortic and first distal sequential anastomosis (arrow). However, a contrast filling of the distal SVG between the first and last distal sequential anastomosis confirms patency in this section of the SVG

The eSVS® mesh (Kips Bay Medical, Inc., Minneapolis, USA) is a flexible extravascular nickel-titanium mesh designed to reinforce SVGs exposed to the higher arterial pressure in CABG [5]. Its preventive effect on reactive intimal hyperplasia was demonstrated in primates and after CE certification in May 2010 the device was released for implantation in humans [6]. Although long-term patency is not yet available first safety reports and short-term results have been recently published [7, 8]. Genoni et al. stated a safe application of the device in 20 patients operated off-pump to receive an eSVS® mesh supported SVG to the right coronary artery [7]. The patency rate 5 days after surgery was 95 %. Klima et al. recently published a 7-months cCTA follow-up in 12 patients after on-pump non-sequential eSVS® mesh supported SVG to both sided coronaries with a patency rate of 92 % [8]. Both studies describe a safe application of the eSVS® mesh and reveal satisfying patency rates when compared to those of non-meshed SVGs [1].

This study presents for the first time one-year patency rates of eSVS® mesh supported SVGs in 19 patients including on- and off-pump revascularisation of the left and right coronary system, sequential and single distal anastomoses and provides information about the vein harvest technique, site, quality and the coronary run-off.

## Methods

### Patients and baseline characteristics, data collection

From June 2010 until June 2011, 22 consecutive patients receiving an eSVS® (Kips Bay Medical, Inc., Minneapolis, USA) meshed saphenous vein coronary bypass graft (SVG) were prospectively enrolled and data analyzed post-hoc. Graft patency as the main endpoint was evaluated by cCTA one year after surgery. Potential risk factors for graft occlusion were statistically analyzed as secondary endpoints. 3 patients were ruled out due to non-device related in-hospital cardiac (*n* = 1) or post-discharge non-cardiac death prior to the scheduled 1-year follow-up.

The data collection (Tables 1, 2 and 3) consisted of patients’ characteristics (including NYHA class), coronary artery disease and left ventricular function (LVEF), surgery (including perfusion-type, native coronary run-off, technique of proximal anastomosis, graft harvest technique / site and quality, mesh sizes, intra-operative transit time flow-measurement, device related complications) and postoperative follow-up (including MACCE). Clinical and cCTA follow-up was provided one year after surgery.

**Table 1:** Patients’ characteristics, surgery, follow-up and mortality

Patients (n) exclusive 3 non-device related deaths	19	
Gender (n; male)	16	
Age (years; mean ± std; min.-max.)	70.4 ± 9.5 (51–81)	
Body weight (kg; mean ± std)	80.1 ± 15.0	
Bodymassindex (kg/m^2^; mean ± std)	28.2 ± 5.3	
NYHA / CCS classification (n; %)	NYHA	CCS
I	13 (68.4 %)	2 (10.5 %)
II	4 (21.1 %)	10 (52.7 %)
III	2 (10.5 %)	5 (26.3 %)
IV	0	2 (10.5 %)
Left ventricular ejection fraction (%; mean ± std)	49 ± 11	
CAD in 1° relative (n; %)	17 (89.5 %)	
Arterial hypertension (n; %)	18 (94.7 %)	
Diabetes (n; %; on insuline/oral antidiabetics/diet)	0 (0 %) / 2 (10.5 %)/6 (31.5 %)	
Dyslipidemia (n; %)	16 (84.2 %) + 1 (5.3 %) unknown	
Smoking (n; %; current/former/never)	2 (10.5 %)/13 (68.4 %)/4 (21.1 %)	
Previous myocardial infarction (n; %)	13 (68.4 %) + 5 (26.3 %) no + 1 (5.3 %) unknown	
Previous cerebrovascular event (n; %)	2 (10.5 %) (TIA only)	
Previous CABG (n; %)	0 (0 %)	
Revascularisation (n;%; OPCAB/ECC/MECC)	11 (57.9 %)/5 (26.3 %)/3 (15.8 %)	
Extracorporeal circulation time (min; mean ± std)^1)^	113.9 ± 35.5 (n = 8)	
Cross clamp time (min; mean ± std)^1)^	79.9 ± 25.3 (n = 8)	
Days on intensive care unit (mean ± std)	2.3 ± 1.3	
In-hospital days (mean ± std)	10.3 ± 4.7	
Directly device-related complications (n)	0	
MACCE (n; %)	1 (5.3 %) (Stroke with paraparesis, complete remission)	

*NYHA*: New York Heart Association, *CCS*: Canadian Cardiovascular Society, *CAD*: Coronary arterial disease, *TIA*: Transient ischemic attack, *CABG*: Coronary artery bypass grafting, *OPCAB*: Off-pump coronary artery bypass, *ECC*: Extracorporeal circulation, *MECC*: Minimal extracorporeal circulation, *MACCE*: Major adverse cardiac and cerebrovascular event, 1) including concomitant aortic valve replacement

**Table 2:** Distal anastomoses, transit time flow-measurement (TTFM) and one-year graft patency by cCTA

*Graft*	*Distal Anastomosis*	*Number (n)*	*Mean Flow by TTFM (ml/min)*	*Pulsatility Index (TTFM)*	*Patent Grafts (n)*	*Occluded Grafts (n)*
*Left Coronary System*						
LIMA	Single	15	40 ± 32.4	2.0 ± 0.6	15	0
	Jump	2^1)^	39 ± 0.0	2.2 ± 0.8	2	0
RIMA	Single	2	58 ± 14.1	1.4 ± 0.6	2	0
	Jump	2^2)^	29 ± 8.5	1.3 ± 0.4	2	0
RADIAL A.	Single	1	19	1.2	1	0
SVG UNMESHED	Jump	4^3)^	37 ± 21.1	2.4 ± 1.0	4	0
SVG MESHED	Jump	10^4)^	73 ± 56.2	1.6 ± 0.4	9	1
TOTAL		36			35	1
*Right Coronary System*						
SVG UNMESHED	Single	2	67 ± 20.5	1.25 ± 0.7	2	0
	Jump	1^5)^	58	2	1	0
SVG MESHED	Single	10	41 ± 28.1	1.5 ± 0.8	6	4
	Jump	1^6)^	78	2	1	0
Total		14			10	4

*TTFM*: Transit time flow-measurement, LIMA: *Left* internal mammary artery, *RIMA*: Right internal mammary artery, Radial A. : Left radial artery, *SVG*: Saphenous vein graft, 1) 5) and 6) All single jump with 2 distal anastomoses, 2) RIMA as a free graft single jump with 2 distal anastomoses (n = 2), 3) Single jump with 2 distal anastomoses (n = 4) and double jump with 3 distal anastomoses (n = 1), 4) Single jump with 2 distal anastomoses (n = 9) and double jump with 3 distal anastomoses (n = 1), 6) Single jump with 2 distal anastomoses (n = 1)

**Table 3:** Venous graft quality, mesh-size, surgery, transit time flow measurement (TTFM), coronary status in occluded grafts (*n* = 5)

Graft No.	1	2	3	4	5
Graft - Target Coronary Vessel	V-RIVP	V-RIVP	V-RCA	V-RCA	V-D-(M)
SVG Harvest technique (Endoscopic: E, Open: O)	E	E	O	E	E
SVG Harvest site (magna: m, parva: p)	m	m	p	m	m
SVG Varicosis^1)^	Yes	Yes	No	No	Yes
SVG Quality^2)^	Moderate	Poor	Poor	Poor	Moderate
SVG Mesh size (mm)	3.5	4.0	4.5	4.0	4.0
Perfusion: OPCAB, ECC, MECC	OPCAB	MECC	ECC	OPCAB	ECC
Aorta: Clamping^3)^ (C), Heartstring (H)	C	C	C	H	C
Native Coronary run-off	Poor: Calcified	Moderate	Poor: Calcified	Moderate	Moderate
Redo Proximal anastomosis (n)	0	0	0	0	0
Redo Distal anastomosis (n)	0	0	0	0	0
TTFM: Flow (ml/min)	47	21	30	15	214
TTFM: Pulsatility index	1.2	0.9	0.8	1.0	1.3

*V*- : « Veingraft to… », *RIVP*: Ramus interventricularis posterior, *RCA*: Right coronary artery, *D*: Ramus diagonalis, *M*: Ramus marginalis, *SVG*: Saphenous vein graft, *E*: Endoscopic saphenous vein harvesting, *O*: Open saphenous vein harvesting, *m*: Vena saphena magna, p: Vena saphena parva, *C*: Anterior tangential sideclamping of the ascending aorta, *H*: HEARTSTRING III Proximal Seal System (Maquet, Holding B.V. & Co. KG., Gossau, Switzerland), *TTFM*: Transit time flow-measurement, 1) Signs of varicosis such as blow-outs, focally thinned out vein graft wall, dilated veingraft > 4.5 mm over > 1/3 of graft length, 2) Vein graft quality according to presence of side branch tears, wall thickness, signs of varicosis such as blow-outs, thinned out vein graft wall and dilated graft > 4.5 mm diameter over > 1/3 of graft length, 3) Partial side clamping of the ascending aorta

### The device: the eSVS® mesh

The eSVS® mesh (External Saphenous Vein Support Device, Kips Bay Medical, Inc., Minneapolis, USA) is a highly flexible, semi-compliant, kink-resistant extravascular tubular prosthesis fabricated of knitted nickel-titanium (nitinol) wire designed to improve autologous vein graft patency in human CABG. The device was CE certified in May 2010 and is released for CABG in humans ever since.

### Anesthesia, Surgery and Procedural Characteristics

All patients received general anesthesia and continuous monitoring by 2-lead electrocardiography (II, V5), pulse-oxymetry and invasive measurement of arterial blood and central venous pressure. Transesophageal echocardiography (TEE) was performed throughout the procedure. Antibiotic prophylaxis (Cefuroxim) was administrated within 30 min prior to skin incision.

All patients were subjected to coronary artery bypass grafting (CABG) with the following revascularisation techniques: Off-pump CABG (OPCAB) and conventional (ECC)/minimal extra corporeal circulation (MECC). Each patient received one eSVS® meshed SVG either to the left or right coronary system grafted either to one single coronary vessel (one single distal anastomosis) or to two or more coronaries (sequential distal anastomoses). The target vessel was selected individually to cover all aspects of contemporary revascularisation (left/right sided, single/sequential grafts) in this patient group. Decision was based on preoperative angiography and intraoperative assessment of the target vessels. A stenosis of > 75 % was considered indispensable for being grafted. The left anterior descending artery (LAD) was routinely grafted using the left internal mammary artery (LIMA). Coronary run-off was classified as poor (calcified vessel AND diameter ≤ 1.5 mm), moderate (calcified vessel OR diameter ≤ 1.5 mm) or good (not calcified vessel AND diameter > 1.5 mm). The residual coronaries with significant stenosis (>75 %) were grafted using the left or right internal mammary artery (LIMA or RIMA), the radial artery or an unmeshed SVG.

Saphenous vein harvesting was routinely performed endoscopically by an experienced resident (>100 harvests) using the ClearGlide (Sorin, Milano, Italy) endoscopic harvest system. A preoperative duplex sonographic vein mapping allowed choosing the harvest site (lower leg or thigh) with the best vein quality. In case of harvesting multiple vein grafts the segment with the superior quality with regards to diameter, lack of varicosis and blow-outs was reserved for meshing. In two cases of absent great saphenous veins (magna) after stripping the minor saphenous vein (parva) was harvested in an open technique with skin bridging. Prior to meshing all side branches were ligated using Prolene 6/0 and residual branch stump tissue resected in order not to provoke intraluminal restriction inside the mesh. Mesh deployment was provided as previously described [5]. Briefly, the appropriate mesh size (3.5, 4.0 or 4.5 mm) was determined by the provided measuring tool. Graft diameters > 7 mm and < 3.6 mm as well as a double wall thickness >1.4 mm were contraindications to apply the eSVS® mesh. The mesh was attached to the SVG by fibrin tissue glue (Tisseel, Baxter, Vienna, Austria). Both its distal and proximal end was bevelled obliquely and incised at the heel for a few millimetres to provide a cobrahead shape of the graft allowing an unrestricted flow across the anastomosis [5, 9]. In situ graft flows were evaluated by intra-operative TTFM after re-uptake of cardiac function and post extracorporeal circulation. Intra-operative complications such as technical meshing problems or the necessity for redo of anastomoses were recorded.

All patients were transferred to the intensive care unit (ICU) and to the ward subsequently. They all received Aspirin and a statin postoperatively.

### Clinical and cCTA 1-year follow-up and major adverse cerebral and cardiovascular events (MACCE)

In-hospital-follow-up continued until discharge for cardiac rehabilitation and included days on ICU/ward and MACCE ((mortality cardiac, non-cardiac), myocardial infarction, stroke, major bleeding requiring surgical revision, new onset kidney injury and major vascular complication).

Patients alive returned for clinical assessment and cCTA one year (12 ± 0.1 months) after surgery. cCTA was given preference to coronary angiography due to lower radiation exposure, avoiding short-term hospitalization and comparable sensitivity and specificity with regards to graft patency detection [10, 11]. Major principal endpoint was meshed graft patency in the cCTA. Secondary endpoints were symptoms (dyspnea, angina pectoris). Furthermore, potential predictors for meshed SVG occlusion were statistically evaluated.

### Data management and statistical analysis

Data were prospectively collected and analysed post-hoc by an independent statistician. Continuous variables are given as mean and standard deviation, categorical variables as numbers (n) and proportions (%). For the descriptive statistics Fisher’s exact test was used for the categorical variables – considering the small number - and T-tests for the continuous variables followed by a non-parametric analysis (Median test). All p values are two-sided and p < 0.05 was considered statistically significant. Analyses have been performed using SPSS 21 (IBM).

### Ethical considerations

The study was approved by the local Independent Ethics Committee and conducted according to Good Clinical Practice (International Conference on Harmonisation) and to the Declaration of Helsinki.

All authors had unlimited access to the complete data set and take responsibility for its integrity. All authors have read and agreed to the manuscript as written.

## Results

### Patients and baseline characteristics

A total of 22 patients received an eSVS® mesh from 06/2010 to 06/2011 (with subsequent one-year cCTA in 19 patients). The eSVS® mesh implantation was successful in all patients. Patients’ baseline characteristics are shown in Table 1.

### Excluded patients

Three patients died prior to 1-year follow-up control and hence were excluded from analysis.

Patient 1 had off-pump revascularisation (sequential LIMA to diagonal branch and LAD, unmeshed sequential SVG to marginal branch I and II, meshed SVG to posterior descending artery (PDA)). On ICU the patient was resuscitated due to sudden pulseless electrical activity (PEA) and had its chest reopened. Though initially stabilized by means of extracorporeal membrane oxygenation (ECMO) the patient finally died due to biventricular heart failure. A device related cardiac failure was excluded by TTFM which revealed excellent flows in all bypass grafts. Patient 2 was revascularized with MECC support (LIMA to LAD, meshed SVG to PDA, unmeshed sequential SVG to diagonal and marginal branches I and II). Cause of death is unknown, however autopsy revealed patent bypass grafts. Patient 3 was revascularized with ECC support (RIMA to right coronary artery (RCA), meshed sequential SVG to intermediate and marginal branches) and mechanical valve replacement. The Patient died 1 year after surgery due to a myelodysplastic syndrome and subsequent sepsis.

### Surgery

Perfusion technique and time of surgery/crossclamping are given in Table 1. Every patient received a meshed SVG. All meshes were deployed safely and implantation was uneventful in all patients. Subsequent intra-operative TTFM revealed an unrestricted patency with good graft flows (59.8 ± 46.7 ml/min) and pulsatility indices (1.6 ± 0.6) in all meshed grafts. Implanted mesh sizes were 3.5 mm (*n* = 3), 4.0 mm (*n* = 13) and 4.5 mm (*n* = 3). There was no intra-operative complication.

### Graft patency and clinical follow-up

There was no directly device-related postoperative complication. One patient developed a stroke with paraparesis with complete remission until discharge. No other MACCE was recorded (excluding one non device-related cardiac in-hospital and two non-device related non-cardiac post-discharge deaths: excluded prior to analysis).

Grafts and target coronary vessels, number of single and sequential distal anastomoses, TTFM flows and pulsatility indices as well as 1-year cCTA graft patency are grouped and summarized in Table 2 for the right and left coronary system, separately. 2.6 ± 0.5 grafts respectively 3.7 ± 0.8 anastomoses were performed per patient. All arterial and unmeshed SVGs were patent in 1-year cCTA. Among the eSVS® supported SVGs four with a single distal anastomosis to the right coronary system were completely occluded and one sequential graft with two distal anastomoses to the left coronary system was partially occluded between the proximal and first distal anastomosis. The overall patency of distal anastomoses was 85 % (*n* = 28/33) and overall graft patency was 76 % (*n* = 16/21). At 1-year follow-up all patients were asymptomatic.

### Risk analysis for graft occlusion

Details of occluded grafts (*n* = 5) are listed in Table 3 and risk evaluation of potential risk factors for meshed graft occlusion are shown in Table 4. Briefly, the intraoperative pulsatility index (PI) evaluated by TTFM had a significant impact on postoperative graft occlusion. PI in occluded grafts (1.0 ± 0.2) had been significantly lower than in non-occluded grafts (1.7 ± 0.6). Furthermore, poor vein quality (no better arterial or venous grafts available) was almost a significant factor related to meshed graft occlusion. No other risk factor for meshed SVG occlusion reached statistical significance despite a predilection for the right coronary system (n = 4/5).

**Table 4:** Evaluation of Potential Risk Factors for eSVS® mesh supported saphenous vein grafts

Risk Factor	Patent	Occluded	P-Value
Gender (n; %; male)	11 (68.8)	5 (31.3)	1.00
Age (years; mean ± std)	72.4 ± 8.2	65 ± 11.8	0.14
CAD in 1° relative (n; %)	13 (76.5)	4 (23.5)	0.47
Arterial hypertension (n; %)	14 (77.8)	4 (22.2)	0.26
Diabetes (n; %)	7 (87.5)	1 (12.5)	0.34
Dyslipidemia (n; %)	12 (75)	4 (25)	0.62
Smoking (n; %; current/former/never)	2(100)/10(76.9)/2 (50)	0 / 3 (23.1)/2 (50)	0.56
Previous myocardial infarction (n;%)	9 (69.2)	4 (30.8)	1.00
LVEF (%; mean ± std)	50.8 ± 12.9	44.8 ± 5	0.13
SVG Harvest technique: endoscopic	13 (76.5)	4 (23.5)	0.47
(n; %)			
SVG Harvest site : magna (n; %)	13 (76.5)	4 (23.5)	0.47
SVG Varicosis (n; %)	9 (75)	3 (25)	1.00
SVG Quality	1 (25)/10 (83.3)/3 (100)	3 (75)/2 (16.7)/0	0.06
(n; %; poor/moderate/good)			
Mesh size (n; %; 3.5 mm/4.0 mm/4.5 mm)^1)^	3 (75)/9 (75)/2 (66.7)	1 (25) / 3 (25)/1 (33.3)	1.00
Perfusion (n; %; OPCAB/ECC/MECC)	9 (81.8)/3 (60)/2 (66.7)	2 (18.2)/2 (40)/1 (33.3)	0.79
Aorta (n; %; Clamping/Heartstring)	4 (50)/9 (90)	4 (50)/1 (10)	0.16
Coronary system (n; %; left/right)	7 (87.5)/7 (63.6)	1 (12.5)/4 (36.4)	0.34
Coronary run-off (n; %; poor/moderate/good)^2)^	1(33.3)/9 (75)/4 (100)	2 (66.7)/3 (25)/0	0.12
Jumping graft (n; %)	8 (88.9)	1 (11.1)	0.30
TTFM: Flow (ml/min; mean ± std)	57.8 ± 28.9	64.4 ± 83.9	0.30
TTFM: Pulsatility index (mean ± std)	1.7 ± 0.6	1.0 ± 0.2	0.03

*CAD*: Coronary arterial disease, *LVEF*: Left ventricular ejection fraction, *SVG*: Saphenous vein graft, *OPCAB*: Off-pump coronary artery bypass, *ECC*: Extracorporeal circulation, *MECC*: Minimal extracorporeal circulation, 1) Diameter, 2) For definition of run-off see “methods” section, TTFM: Transit time flow measurement

## Discussion

One year after a safe implant of 19 eSVS® meshes without intra- or postoperative device-related complications all patients were asymptomatic and 76 %/85 % of grafts/anastomoses were patent. Including off- and on-pump, sequential as well as right and left sided revascularisation we found a prescriptive predilection for meshed graft occlusion to the right coronary, in poor SVG quality and coronary run-off.

The overall patency rate of SVGs is inferior to arterial grafts [1]. Early SVG occlusion occurs due to thrombosis in up to 12 % already within the first six postoperative months and graft patency is affected by narrowing intimal hyperplasia and subsequent atherosclerosis [2, 12]. Therefore, a rising concern for arterial revascularisation has substantially influenced the current guidelines [4]. Nevertheless, SVGs are still essential in absent suitable arterial conduits if they are the only available graft material. In coronary stenoses < 90 % they are even favourable to arteries as arterial spasms and a high concurring coronary flow might lead to early graft occlusion [3, 4]. The eSVS® mesh externally reinforces SVGs to reduce circumferential stress subsequently leading to endothelial damage favouring SVG disease [2]. Hence it should conceptionally improve SVG patency.

First animal eSVS® mesh implants demonstrated this preventive effect on early (12 weeks) intimal hyperplasia in primates. Schoettler, Genoni and Klima et al. published first in human short and intermediate-term patency rates [6–8, 13]. Schoettler et al. state a detrimental 27.8 % nine-months-patency-rate in meshed against 85.7 % in non-meshed SVGs and substantially questioned the safety of the eSVS® device [13]. Subsiding results of Genoni et al. with a 95 % and Klima et al. with a 92 % eSVS® patency rate disproved Schoettler’s findings [7, 8]. An evident technical implant difference of Schoettler was the lack of forming a cobrahead at the anastomoses leading to a steep angulation comprising the flow across the anastomosis as discussed by Genoni et al. and by us in a short communication [7, 9]. As Emery et al. finally emphasized the importance of forming a cobrahead all our patients were grafted with a cobrahead [5]. Our patency rate of 76 %/85 % (of grafts/anastomoses) is comparable to the literature of unmeshed SVGs despite our 100 % patency of untreated SVGs [1, 14]. On the other hand our meshed SVG patency seems slightly inferior to Genoni’s and Klima’s findings [7, 8]. However, relevant differences in the time-span until follow-up, surgical setup, revascularized coronary targets and the use of sequential anastomoses impedes a direct comparability of these cited papers. Genoni et al. present very short-term results 5 days after surgery without any further published follow-up and focusses exclusively on the right sided and off-pump revascularisation [7]. The coronary run-off (calcification, vessel diameter) is not mentioned which is a known predictive factor for SVG patency [1]. Klima et al. operated only on-pump omitting sequential anastomoses and presents a relatively smaller patient number (n = 12) assessed by cCTA [8]. Klima’s follow-up with 12 out of 21 patients (57.1 %) is relatively low and cCTA control was performed within a variable time-span of 3 to 14 months after surgery. In contrast, the present study comprises various revascularisation techniques (OPCAB, ECC and MECC), includes single and sequential distal anastomoses to both sided coronary targets, respects the SVG quality and coronary run-off, presents the longest cCTA follow-up (12 ± 0.1 months) and encompasses a larger patient collective with a more comprehensive follow-up than Klima et al. [8].

The risk evaluation of the present study adds to the value (Table 4) and statistical analysis was performed with regards to the relatively small patient number. No cardiovascular risk factor (arterial hypertension, diabetes, CAD in 1° relative, etc.) revealed a significant difference in meshed and unmeshed SVG occlusion suggesting a safe application of the eSVS® mesh irrespectively of the patient’s cardiovascular risk profile. Neither the harvest technique nor site significantly influenced meshed SVG occlusion. Although Lopez et al. questioned safety of endoscopic vein harvesting recent studies prove its uncompromised safety in the hands of experienced surgeons [15, 16]. Our high portion of sequential meshed anastomoses confidently ruled out a negative impact on meshed SVG patency. Basically, the peripheral coronary resistance decreases with the number of sequential anastomoses and a better graft flow decreases the risk for graft occlusion. Klima et al. excluded sequentials whereas Genoni et al. did not [7, 8]. We found one meshed SVG partially occluded between the proximal and the first sequential distal anastomosis. As the distal graft was patent we assume a problem at the proximal anastomosis reinforcing the safe use of sequential meshed revascularisation. A non- significant predilection of meshed SVG occlusion to the right coronary system (*n* = 4/5) was observed. Despite an excellent patency-rate exclusively to the right coronary system a direct comparison of Genoni’s data is delicate considering the shorter postoperative follow-up of 5 days and no comparable data of the left sided revascularisation and the coronary run-off [7]. Both Goldmann and Hess et al. evaluated a compromised coronary run-off as well as a right coronary target as significant risk factors for early SVG occlusion [1, 17]. Goldmann et al. even found similar right sided occlusion rates (around 80 % after 1 year) relativating our findings and supporting the theory that predilection to the right side occured despite the lack of statistical significance. The pulsatility index by TTFM reached statistical significance (Table 4 : *p* = 0.03) whereas the flow measure did not. Une et al. associated a TTFM flow < 31 ml/min with significantly worse one-year SVG patency than higher flows in CABG [18]. The adequacy of intraoperative TTFM in meshed SVGs was published by Emery et al. [19]. However, our conflicting association of a low PI and graft occlusion questions its predictive value. Furthermore, the use of fibrin sealant and the technique of meshing the entire SVG might be causative for meshed graft occlusion, too. Emery et al. recommends a preferably thin external layer of fibrin sealant to reliably fix the mesh on the SVG [5]. This seems essential at the anastomoses as the loops of the nitinol mesh are partially opened when cutting the meshed graft to its appropriate length. Hereby, open loops can easily get to lie intraluminally with a subsequent thrombogenic effect leading to graft occlusion. Despite this protective impact the direct effect of fibrin sealant on meshed SVG patency remains unclear. Therefore, other devices such as VEST (Venous External Scaffolding Technique, Vascular Graft Solutions Ltd., Tel Aviv, Israel) suggest a mesh-free SVG at the site of anastomosis.

The main limiting factor of this study is the relatively low number of patients. Hence a careful interpretation of the data is mandatory although it was considered in the statistical analysis. An adequate follow-up and patient volume, safe sequential off- and on-pump left and right sided revascularisation reinforce this study towards the background of the current state of recent literature.

## Conclusions

In conclusion we safely implanted 19 eSVS® meshes without directly device-related complications with an overall one-year SVGs/anastomoses patency-rate of 76 %/85 % comparable to the literature of meshed and non-meshed SVGs. Neither cardiovascular risk profile, revascularisation technique, vein harvest site and technique, coronary target side and run-off nor sequential or single distal anastomoses were significant predictive factors for graft occlusion. On the other hand, descriptive predilection of the right coronary system, vein varicosis and poor coronary run-off can serve as soft indicators for early graft occlusion in eSVS® supported vein grafts.

However, graft patency of meshed veins (76 %) was inferior to non-meshed (100 %) or arterial grafts (100 %). Thus our mid-term data do not sustain the concept of improving vein graft patency by external reinforcing with the eSVS® mesh. Further long-term follow-up is warranted.

## References

[1] Goldman S, Zadina K, Moritz T, Ovitt T, Sethi G, Copeland JG (2004). Long-term patency of saphenous vein and left internal mammary artery grafts after coronary artery bypass surgery: results from a Department of Veterans Affairs Cooperative Study. J Am Coll Cardiol.

[2] Kim FY, Marhefka G, Ruggiero NJ, Adams S, Whellan DJ (2013). Saphenous vein graft disease: review of pathophysiology, prevention, and treatment. Cardiology in review.

[3] Jeong DS, Kim YH, Lee YT, Chung SU, Sung K, Kim WS (2013). Revascularization for the right coronary artery territory in off-pump coronary artery bypass surgery. Ann Thorac Surg.

[4] Kolh P, Windecker S, Alfonso F, Collet JP, Cremer J, Falk V (2014). 2014 ESC/EACTS Guidelines on myocardial revascularization: The Task Force on Myocardial Revascularization of the European Society of Cardiology (ESC) and the European Association for Cardio-Thoracic Surgery (EACTS)Developed with the special contribution of the European Association of Percutaneous Cardiovascular Interventions (EAPCI). Eur J Cardiothorac Surg.

[5] Emery RW, Solien E, Jamieson SW (2012). Implantation of the eSVS Mesh. Innovations (Philadelphia, Pa).

[6] Zilla P, Human P, Wolf M, Lichtenberg W, Rafiee N, Bezuidenhout D (2008). Constrictive external nitinol meshes inhibit vein graft intimal hyperplasia in nonhuman primates. The Journal of thoracic and cardiovascular surgery.

[7] Genoni M, Odavic D, Loblein H, Dzemali O (2013). Use of the eSVS Mesh: external vein support does not negatively impact early graft patency. Innovations (Philadelphia, Pa).

[8] Klima U, Elsebay AA, Gantri MR, Bangardt J, Miller G, Emery RW (2014). Computerized tomographic angiography in patients having eSVS Mesh(R) supported coronary saphenous vein grafts: intermediate term results. Journal of cardiothoracic surgery.

[9] Inderbitzin DT, Matt P, Eckstein FS, Reuthebuch O (2011). eComment: external nitinol meshing of venous coronary artery bypass grafts: is safety of application really in doubt?. Interact Cardiovasc Thorac Surg.

[10] Achenbach S, Narula J (2011). Coronary CT angiography: from sensitivity to specificity. JACC Cardiovascular imaging.

[11] Schueler S, Walther S, Schuetz GM, Schlattmann P, Dewey M (2013). Methodological quality of diagnostic accuracy studies on non-invasive coronary CT angiography: influence of QUADAS (Quality Assessment of Diagnostic Accuracy Studies included in systematic reviews) items on sensitivity and specificity. European radiology.

[12] Fitzgibbon GM, Kafka HP, Leach AJ, Keon WJ, Hooper GD, Burton JR (1996). Coronary bypass graft fate and patient outcome: angiographic follow-up of 5,065 grafts related to survival and reoperation in 1,388 patients during 25 years. J Am Coll Cardiol.

[13] Schoettler J, Jussli-Melchers J, Grothusen C, Stracke L, Schoeneich F, Stohn S (2011). Highly flexible nitinol mesh to encase aortocoronary saphenous vein grafts: first clinical experiences and angiographic results nine months postoperatively. Interact Cardiovasc Thorac Surg.

[14] FitzGibbon GM, Leach AJ, Kafka HP, Keon WJ (1991). Coronary bypass graft fate: long-term angiographic study. J Am Coll Cardiol.

[15] Lopes RD, Hafley GE, Allen KB, Ferguson TB, Peterson ED, Harrington RA (2009). Endoscopic versus open vein-graft harvesting in coronary-artery bypass surgery. The New England journal of medicine.

[16] Dacey LJ (2012). Endoscopic vein-graft harvest is safe for CABG surgery. Jama.

[17] Hess CN, Lopes RD, Gibson CM, Hager R, Wojdyla DM, Englum BR (2014). Saphenous Vein Graft Failure After Coronary Artery Bypass Surgery: Insights From PREVENT IV. Circulation.

[18] Une D, Deb S, Chikazawa G, Kommaraju K, Tsuneyoshi H, Karkhanis R (2013). Cut-off values for transit time flowmetry: are the revision criteria appropriate?. Journal of cardiac surgery.

[19] Emery RW, Solien E (2013). Intraoperative transit-time flow measurement is not altered in venous bypass grafts covered by the eSVS mesh. Innovations (Philadelphia, Pa).

